# Rapid adaptation to microgravity in mammalian macrophage cells

**DOI:** 10.1038/s41598-017-00119-6

**Published:** 2017-02-27

**Authors:** Cora S. Thiel, Diane de Zélicourt, Svantje Tauber, Astrid Adrian, Markus Franz, Dana M. Simmet, Kathrin Schoppmann, Swantje Hauschild, Sonja Krammer, Miriam Christen, Gesine Bradacs, Katrin Paulsen, Susanne A. Wolf, Markus Braun, Jason Hatton, Vartan Kurtcuoglu, Stefanie Franke, Samuel Tanner, Samantha Cristoforetti, Beate Sick, Bertold Hock, Oliver Ullrich

**Affiliations:** 1 Institute of Anatomy, Faculty of Medicine, University of Zurich, Winterthurerstrasse 190, 8057 Zurich, Switzerland; 2 Department of Machine Design, Engineering Design and Product Development, Institute of Mechanical Engineering, Otto-von-Guericke-University Magdeburg, Universitätsplatz 2, 39106 Magdeburg, Germany; 3 Space Life Sciences Laboratory (SLSL), Kennedy Space Center, 505 Odyssey Way, Exploration Park, FL 32953 United States of America; 4 Institute of Physiology, Faculty of Medicine, University of Zurich, Winterthurerstrasse 190, 8057 Zurich, Switzerland; 5 Airbus Defence and Space, GmbH, Claude-Dornier-Strasse, 88090 Immenstaad, Germany; 6 German Aerospace Center (DLR), Space Agency, Königswinterer Strasse 522-524, 53227 Bonn, Germany; 7 European Space Agency (ESA), Keplerlaan 1, 2201 AZ Noordwijk, Netherlands; 8 Zurich Center for Integrative Human Physiology (ZIHP), University of Zurich, Winterthurerstrasse 190, 8057 Zurich, Switzerland; 9 German Aerospace Center (DLR), Linder Hoehe, 51147 Cologne, Germany; 10 BIOTESC, CC Aerospace Biomedical Science and Technology, Lucerne School of Engineering and Architecture, Technikumstrasse 21, 6048 Horw, Switzerland; 11 Epidemiology, Biostatistics and Prevention Institute (EBPI), University of Zurich, Zurich, Switzerland; 12 Chair of Proteomics and Bioanalytics, Technical University of Munich, Alte Akademie 14, 85354 Freising, Germany

## Abstract

Despite the observed severe effects of microgravity on mammalian cells, many astronauts have completed long term stays in space without suffering from severe health problems. This raises questions about the cellular capacity for adaptation to a new gravitational environment. The International Space Station (ISS) experiment TRIPLE LUX A, performed in the BIOLAB laboratory of the ISS COLUMBUS module, allowed for the first time the direct measurement of a cellular function in real time and on orbit. We measured the oxidative burst reaction in mammalian macrophages (NR8383 rat alveolar macrophages) exposed to a centrifuge regime of internal 0 g and 1 g controls and step-wise increase or decrease of the gravitational force in four independent experiments. Surprisingly, we found that these macrophages adapted to microgravity in an ultra-fast manner within seconds, after an immediate inhibitory effect on the oxidative burst reaction. For the first time, we provided direct evidence of cellular sensitivity to gravity, through real-time on orbit measurements and by using an experimental system, in which all factors except gravity were constant. The surprisingly ultra-fast adaptation to microgravity indicates that mammalian macrophages are equipped with a highly efficient adaptation potential to a low gravity environment. This opens new avenues for the exploration of adaptation of mammalian cells to gravitational changes.

## Introduction

The gravitational force has been constant throughout the 4 billion years of Earth’s evolutionary history^1^ and probably played a crucial role in the evolutionary explosion of organisms^2^. All terrestrial life, including man, has adapted to this fundamental force by developing a number of important features in their composition and functions^3^, whereas changes of the gravitational environment induce strong alterations of human physiological systems, which respond and adapt to the new gravitational environment within hours or weeks^4^. Importantly, microgravity has been demonstrated to have profound effects at the cellular and molecular level, including changes in cell morphology, proliferation, growth, differentiation, signal transduction and gene expression^5, 6^. In spite of the witnessed serious and often disastrous effects on cells, many astronauts have now completed long term stays in space without suffering from any severe health problem associated with the effect of microgravity^4, 6^. Regarding the innate component of the immune response, the magnitude of the change during spaceflight is not great or the pattern of change across various functions is not consistent^6^. This leads to the hypothesis that the cells of the body must have an enormous capacity to adapt to microgravity, be capable of reacting to altered environmental conditions and of restoring cellular functions to a considerable degree. In this regard, isolated cells are an ideal study object for the investigation of the impact of microgravity without any disturbing or masking secondary effects. Some studies reported that key cellular mechanisms are altered in microgravity, followed by an adaptation of the system to the new circumstances by executing countermeasure actions. Such countermeasures include the modification of the cytoskeleton and nuclear morphology, which occurs after 32 hours^7^. It was recommended by the National Academies of Science, Engineering and Medicine of the US to investigate the reversibility of the changes that occur during and after flight more carefully^6^. In our study, we demonstrated for the first time and through real-time measurements on board of the International Space Station (ISS) that mammalian cells have the capacity to adapt to microgravity within seconds.

Three reasons led us to choose the oxidative burst reaction in mammalian macrophages for real-time measurements of cellular effects of gravity on board of the ISS. Firstly, the oxidative burst reaction represents one of the key elements in the innate immune response, and it is the most important barrier against microbes invading the body^8^. Secondly, it is a very ancient part of the immune system. NADPH oxidases, enzymes which transport electrons and generate reactive oxygen species (ROS), were present from the earliest stages of evolution in every type of multicellular life^9^. Thirdly, our previous experiments demonstrated that the oxidative burst reaction is inhibited in microgravity^10, 11^. This fast inhibition facilitates the determination of threshold levels of gravity sensitivity and the investigation of adaptation and re-adaptation effects.

## Results

### Direct evidence of sensitivity to altered gravitational forces through real-time on-orbit measurements

**Figure 1:**
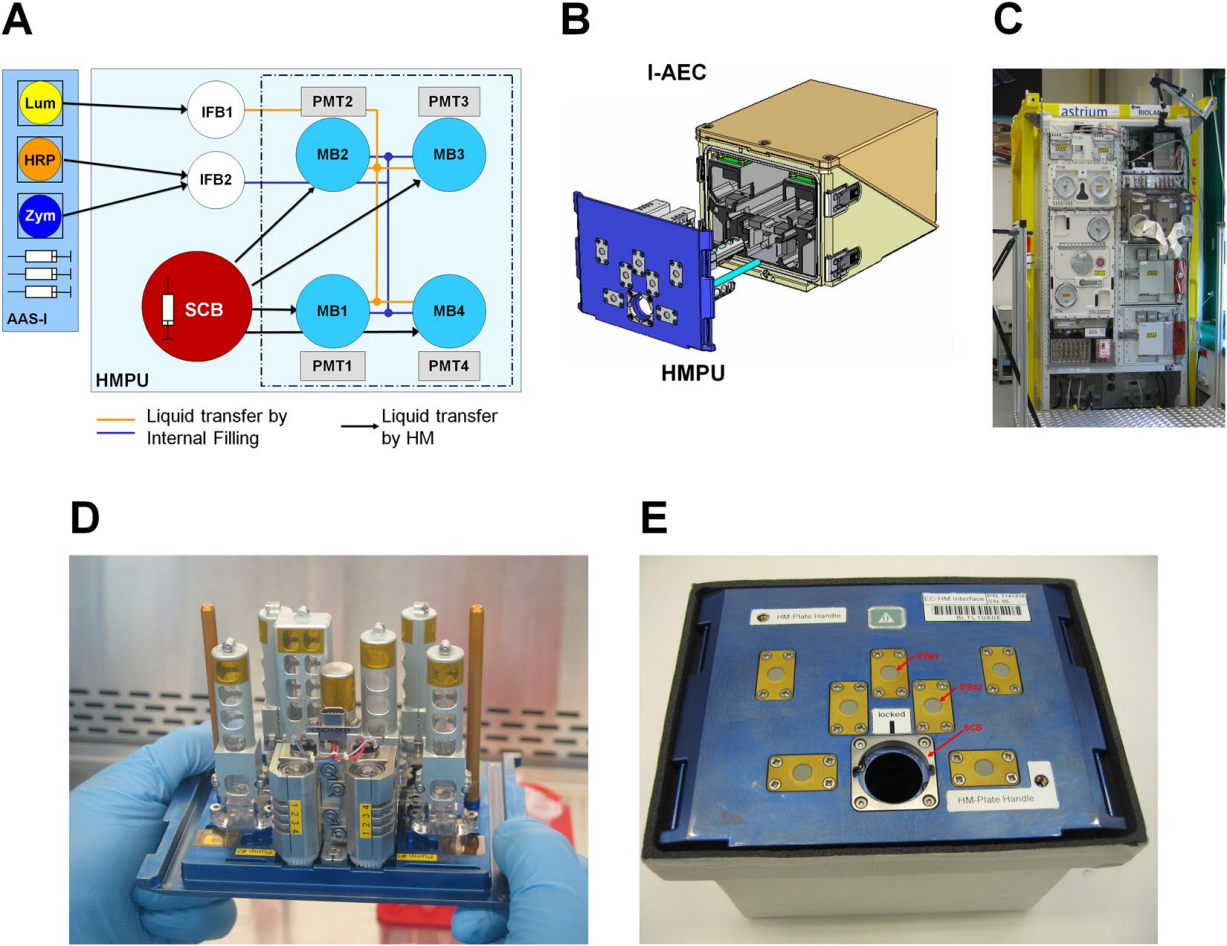
Hardware of the ISS experiment TRIPLE LUX A. (**A**) Experiment system: Production of reactive oxygen species (ROS) were measured by photomultiplier tube (PMT) detected luminescence reaction. The main hardware items are: Stock Culture Bags (SCB) (storable down to −96 °C), Integrated Advanced Experiment Container (I-AEC) containing PMT detector systems with four measurement cuvettes and electronics, Handling Mechanism Plate Unit (HMPU) prepared with cell culture medium (storable down to −20 °C). NR8383 rat macrophage cells in SCBs and selected reagents were launched deep frozen at −80 °C and stored frozen on orbit (MELFI = Minus Eighty degrees Laboratory Freezer for ISS) and in the Biological Experiment Laboratory (BIOLAB) temperature controlled unit at −20 °C until the start of the experiment, respectively. Samples and reagents can be stored for several months on-orbit. SCBs were thawed at ambient temperature (18–28 °C) for 37 min (required for operational safety reasons), then reconstituted at 37 °C for 3 hours (BIOLAB), transferred to the measurement bags (MBs) and incubated with a mixture of zymosan (Zym), horse radish peroxidase (HRP) and luminol (Lum) via the internal filling bags (IFBs) from the automated ambient storage insert (AAS-I). (**B**) Assembled I-AEC with HMPU. (**C**) BIOLAB ground model (Microgravity User Support Center MUSC, DLR). (**D**) HMPU prepared with cell culture medium during integration procedure at Kennedy Space Center. (**E**) Front plate of assembled HMPU with insertion port for SCB and injection ports for the IFBs. All liquid handling was executed by the automated handling mechanism of the BIOLAB.

**Figure 2:**
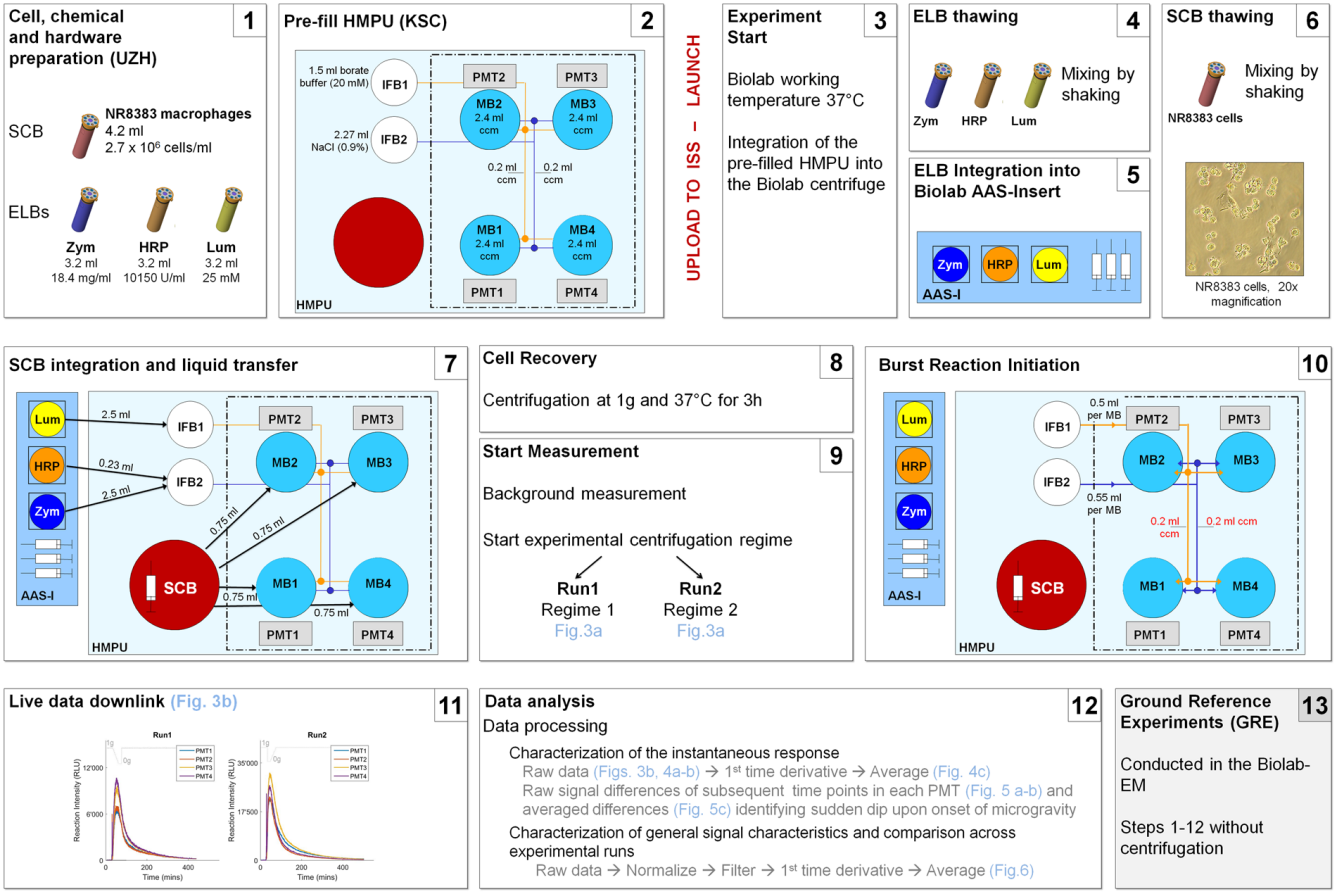
Timeline of the flight (1–12) and ground ref. (13) experiments and data analysis. (1) NR8383 macrophageal cells (4.2 ml, 2.7 × 10^6^ cells/ml) were filled in stock culture bags (SCBs). Cells were stored at −80 °C before, during and after upload to the International Space Station (ISS). Chemicals (luminol (Lum), zymosan (Zym), horse radish peroxidase (HRP)) were prepared, tested for their reactivity, and filled into extra liquid bags (ELBs) (3.2 ml) which were stored at −80 °C before and during the upload to the ISS. On board the ISS ELBs were stored at −20 °C. (2) The two sterile handling mechanism plate units (HMPU) were prefilled before upload. The internal filling bags (IFB) 1 and 2 were filled with 1.5 ml borate buffer and 2.27 ml NaCl, respectively. The four measurement bags (MB1–4) were filled with 2.4 ml of cell culture medium (ccm) each and the tubings with 0.2 ml ccm. The two HMPUs were stored before, during and after upload at 4 °C. (3) The experiment on board the ISS started with pre-warming the BIOLAB Rack to 37 °C. Thereafter, the pre-filled HMPU was integrated into the BIOLAB centrifuge. (4) ELBs were thawed and mixed. (5) The ELBs were implemented into the Automatic Ambient Stowage-Insert (AAS-I). (6) NR8383 cells in the SCB were thawed and mixed. (7) The SCB was integrated into the designated space in the HMPU. Liquids were transferred by the automated handling mechanism (HM) from the AAS-I into IFB1 and IFB2 and from the SCB into MB1-4. (8) Centrifugation at 37 °C for 3 h for cell recovery. (9) Turning-on of the 4 Photomultiplier Tubes (PMTs) and start of the measurement acquisition, experimental centrifugation regimes, and (10) pumping of the liquids from IFB1 and IFB2 into MB1-4 to start the oxidative burst reaction. After injection of the fluids from IFB1 and IFB2, the concentrations in MB1-4 were 0.44 × 10^6^ NR8383 cells per ml, 1.7 mM luminol, 1.1 mg/ml zymosan and 55.85 U/ml HRP. (11) Live data downlink during the experiment. (12) Post flight data analysis. (13) The ground reference experiments (GRE1 and GRE2) were conducted in the BIOLAB engineering model (EM) following the same procedure as the flight experiments (Steps 1–12), except that there was no centrifugation in Steps 8 and 9. The cells recovered for 3 h under Earth 1 g conditions at 37 °C.

**Figure 3:**
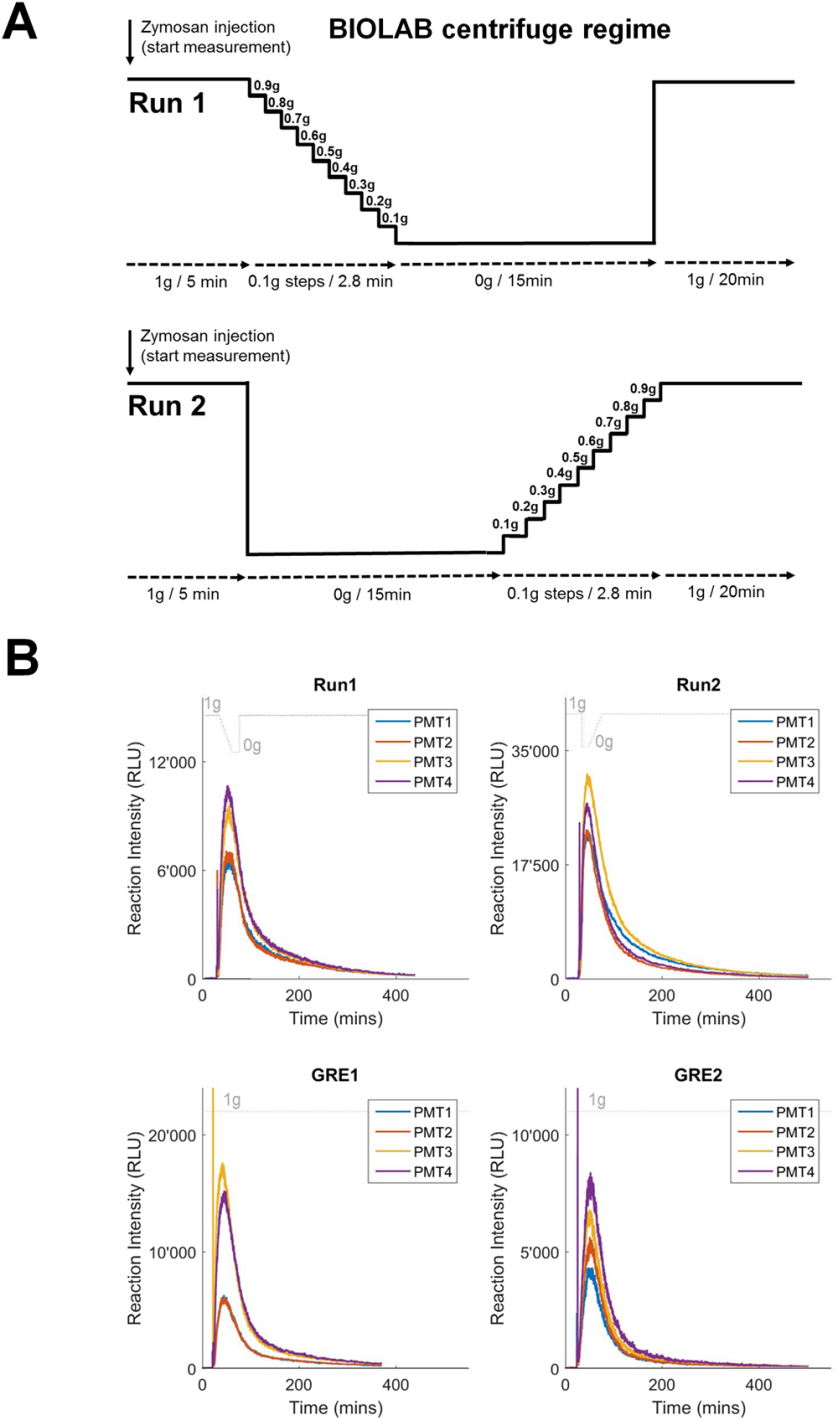
(**A**) BIOLAB centrifuge regimes with step-wise increase or decrease of gravity. Two different centrifuge regimes, one with step-wise increase (from 0 g to 1 g, Run2) and the other with step-wise decrease (from 1 g to 0 g, Run1) of the gravity levels, allowed the determination of gravity thresholds in the mammalian cell system and the investigation of adaptation and re-adaptation effects. The two runs were performed sequentially, each run was subjected to a specific centrifuge regime after the zymosan/horseradish peroxidase injection into the MBs: Run1 started with a 1 g baseline, followed by a step-wise decrease from 1 g to 0 g in 0.1 g/2.8 min steps, a 15 min 0 g phase and finally a rapid increase to 1 g. Run2 started with a 1 g baseline, followed by a 15 min 0 g phase, a step-wise increase from 0 g to 1 g in 0.1 g/2.8 min steps and finally a 1 g phase. The centrifuge transition times of the 0.1 g steps were 2–4 s between 0.1 g and 1 g and 6–9 s between 0 g and 0.1 g, the direct transition time between 1 g and 0 g was 30 s. (**B**) Direct real-time measurements of oxidative burst reaction in NR8383 rat macrophages, expressed in relative light units (RLU). The acceleration profiles (expressed in g) are indicated above each set of curves. Run1 and Run2: On orbit experiments (BIOLAB flight model, ISS) with centrifuge regimes demonstrated in Fig. 3a. GRE1 and GRE2: Ground reference experiments under constant 1 g force (BIOLAB ground model, MUSC/DLR). Photon counts of four parallel experiments (PMT recordings) with a frequency of 1 Hz.

**Figure 4:**
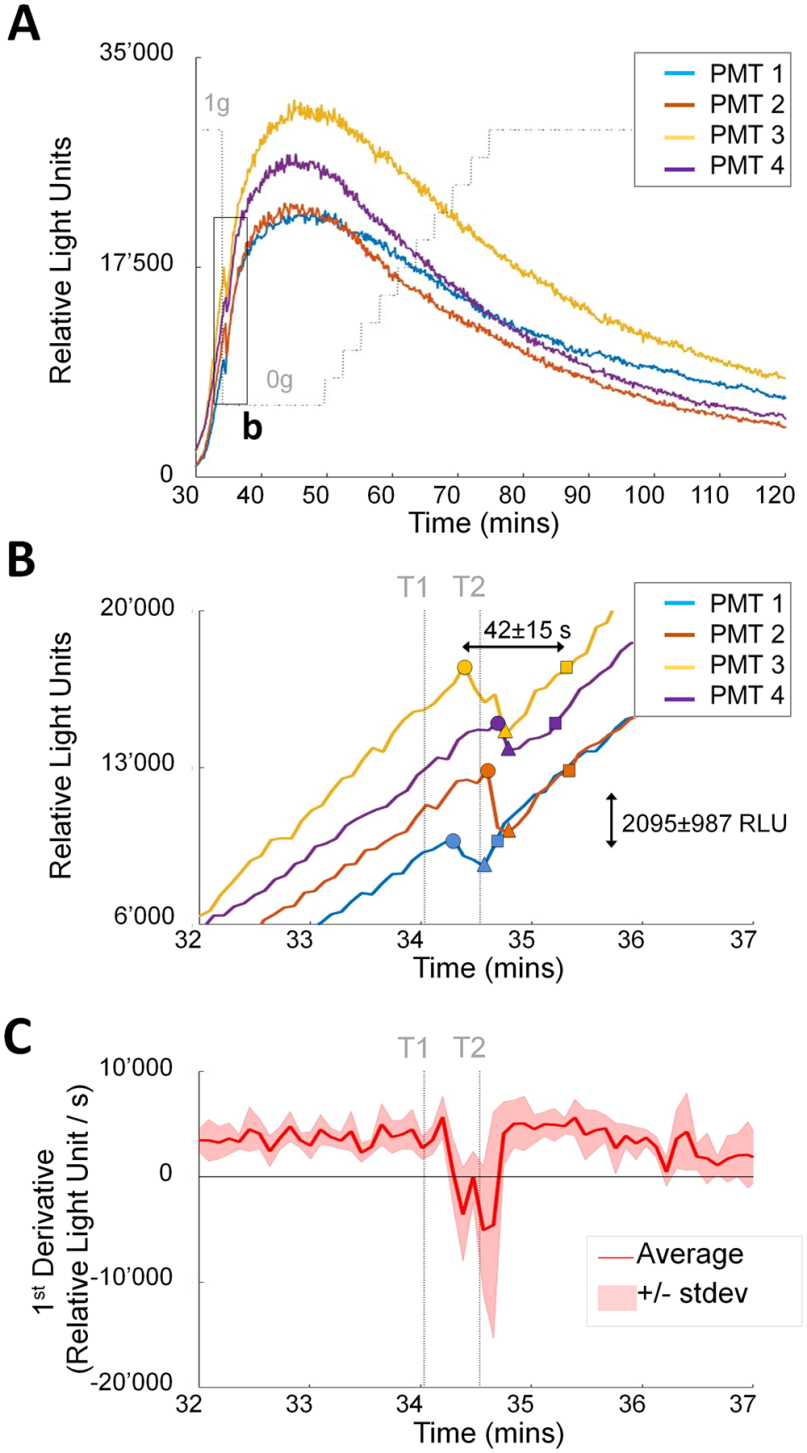
Rapid adaptation to microgravity. (**A**) Raw fluorescent measurements up to 90 mins after zymosan injection. The oxidative burst reaction showed a sharp downward spike (see Fig. 4a) immediately after transition from 1 g to 0 g in all four independent experiments. (**B**) Magnified view of the oxidative burst decline and recovery after 1 g to 0 g transition. Symbols illustrate for each PMT the last measurement before signal decline (circle), local minimum before the onset of the adaptation process (triangles), and the time point at which the signal recovers its initial value (squares). Average amplitude of the downward spike and recovery time were 2095 ± 987 RLU and 42 ± 15 s, respectively. (**C**) Oxidative burst decline and recovery illustrated on the signals’ first time derivative. The thick red line depicts the average of first derivatives of the four independent PMT measurements, while the shaded area shows the standard deviation. All four independent experiments exhibited an immediate inhibitory effect of microgravity on oxidative burst reaction (negative derivative), followed by full adaptation after 42 s +/− 15 s (derivative back to its initial value). T1 = centrifuge stop command, T2 = full stop of the centrifuge 30 s after centrifuge stop command.

We developed the TRIPLE LUX A experiment system, which enables real-time on orbit measurement of oxidative burst reaction in mammalian NR8383 macrophage cells and the following real-time data downlink to Earth (Figs 1 and 2). Two different BIOLAB centrifuge regimes (Fig. 3a), one with step-wise decrease from 1 g to 0 g and sudden increase back to 1 g (Run1) and the other with a sudden decrease from 1 g to 0 g and step-wise increase back to 1 g (Run2), allowed for the determination of gravity threshold levels in the mammalian cell system and immediate 0 g/1 g transitions with subsequent constant gravitational force for the investigation of adaptation effects. After the frozen storage on orbit, cells were thawed and adapted to 1 g for 3 h in the BIOLAB centrifuge (Fig. 2). The thawing and initial 1 g centrifuge regime ensured that the cells were not exposed to 0 g at any time before the experiment. Figure 3b illustrates the raw signals of the two on orbit runs and two ground reference experiments. In all four independent experiments of Run2, the oxidative burst reaction rapidly declined after the transition from 1 g to 0 g (Fig. 4a–c).

### Direct evidence of rapid adaptation to microgravity

**Figure 5:**
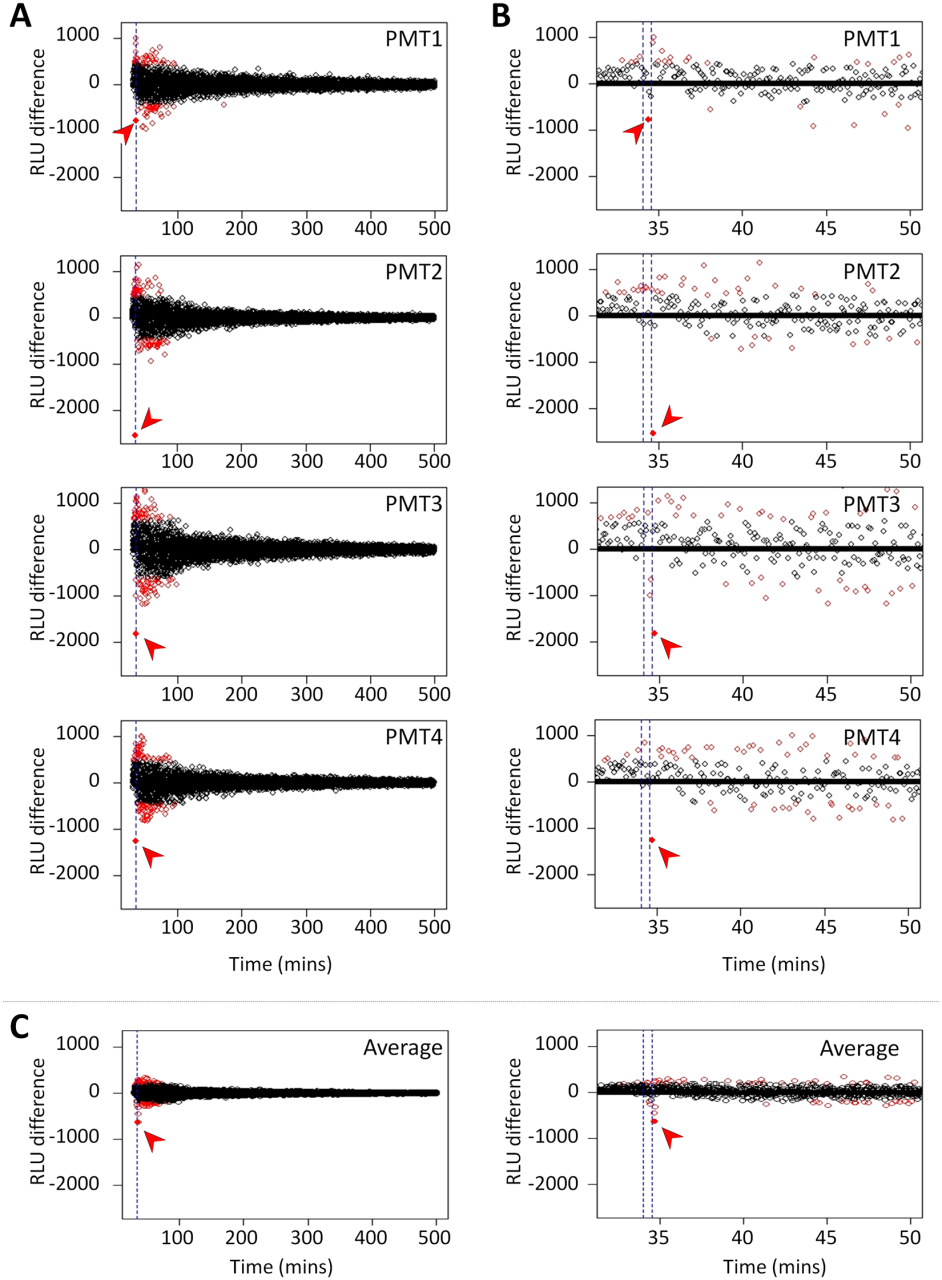
Raw signal differences of consecutive measurement time points as a function of time. (**A**) Course of signal differences over the whole measurement time of 500 minutes observed in PMT1-4. (**B**) Zoom-in into the relevant time frame around the onset of microgravity. (**C**) Average of the four independent signal differences (PMT1-4) over the whole experiment (left) and as zoom-in (right). The blue lines mark the centrifuge stop command at 34.033 min and full centrifuge stop at 34.533 min, respectively. Red points mark significant deviation from a normal variation around zero (quantified by a test on z-scores on a significance level of alpha = 10^−10^). The dip of the raw signal upon onset of microgravity corresponds to the most extreme signal change (marked by a red arrow) and occurs around the time point of centrifuge stop.

The decline of the oxidative burst reaction started 30.9 s +/− 10.5 s after the centrifuge stop command. At this point the residual gravitational force was between 1.05 × 10^−1^ g and 5.98 × 10^−6^ g (Fig. 4b). The interval between the BIOLAB centrifuge stop command at 34.033 min and full centrifuge stop at 34.533 min after the start of the measurement (see Fig. 2 step 9) was 30 s (T1 and T2 in Fig. 4b and blue lines in Fig. 5). Therefore, the oxidative burst declined immediately after a microgravity environment was achieved. In previous parabolic flight experiments we found an immediate (timeframe of a second) decline of the oxidative burst reaction with the onset of microgravity^9^, which could be therefore confirmed on orbit. The functional onset of the adaptation processes (=minimum of the oxidative burst curve, triangles in Fig. 4b) was measured after 14 s ± 7 s and the oxidative burst recovered to its initial value after 42 s ± 15 s (squares in Fig. 4b) in the microgravity environment. The burst signal decrease by a mean of 2095 RLU upon microgravity onset was found to be highly significant and the strongest signal jump over 500 minutes of measurement. Figure 5a shows the signal differences between consecutive time points as recorded by the four photomultiplier tubes (PMTs, one per individual experiment) of Run2. This signal difference provides a measure of the raw signal changes during Run2. The most extreme signal change over the entire acquisition period (consisting of 128’199 signal changes) was identified at time points 34.66 min, 34.7 min, and 34.72 min, for PMT 2, 3 and 4, respectively (red arrows in Fig. 5). These extreme signal changes correspond to the dip noted on the raw signal (Fig. 4) and correlate exactly with the centrifuge stop and the transition from 1 g to 0 g. For the PMT1 measurement, there is also a strong signal change at 34.36 min correlating with the onset of microgravity but it was less extreme than in the other 3 PMTs (Fig. 5a–c). However, also for the average of the signal differences across all four PMT measurements the most extreme signal change over the whole measurement time of 500 minutes was found at 34.66 min and is perfectly in the timeframe of the onset of microgravity (Fig. 5c).

### Indication of thresholds in the sensitivity to gravity

**Figure 6:**
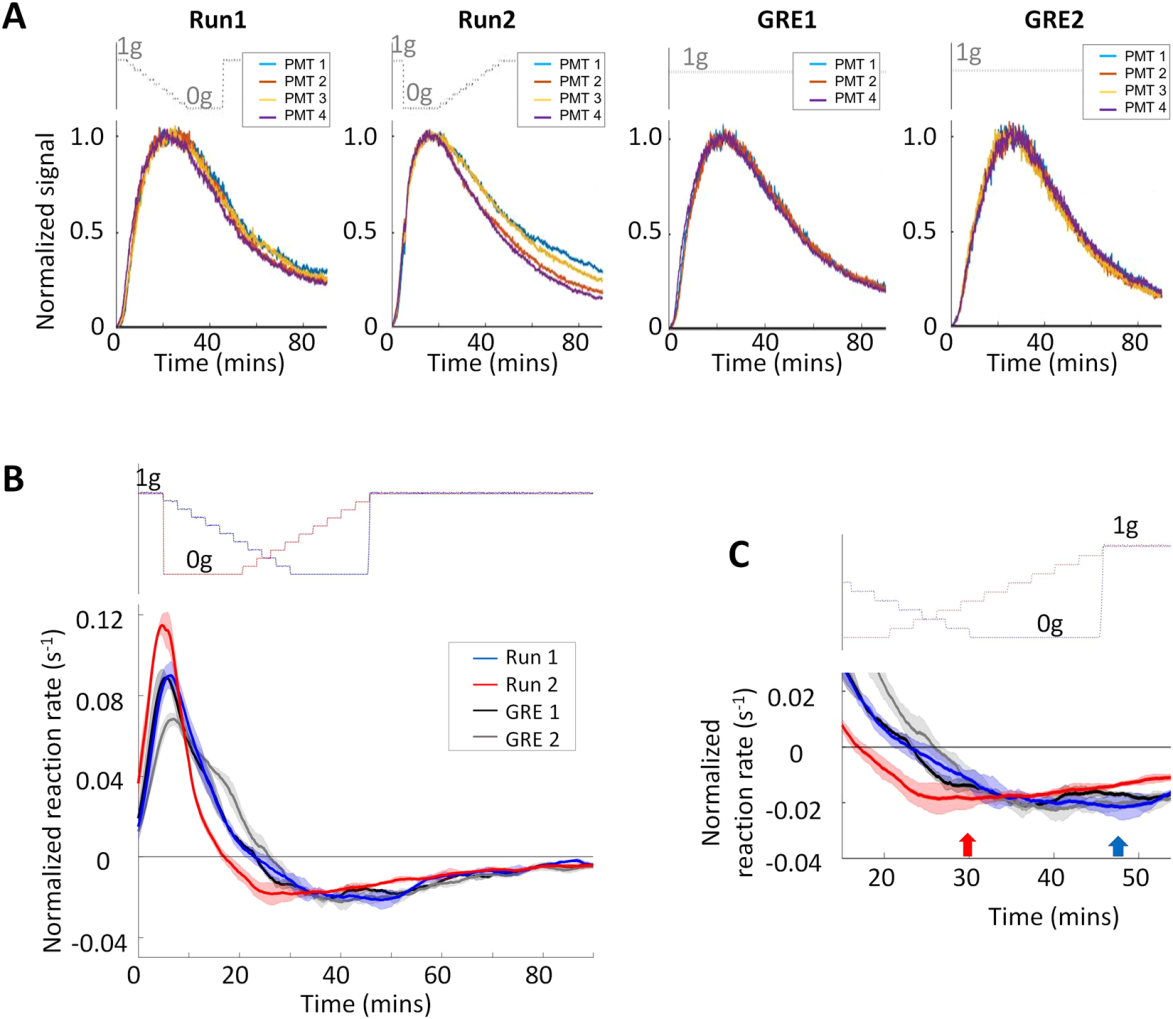
Comparison of the oxidative burst reaction rate across experimental conditions. (**A**) Synchronized and normalized signals. To account for differences in reaction initiation times and nominal signal intensity variations, the raw PMT measurements were synchronized in time, setting the time of zymosan injection as t = 0, and normalized by the peak signal intensity. For GRE1, PMT3 was a clear outlier and removed from the analysis. (**B**) Mean reaction rates over the first 90 min. The individual normalized signals were filtered to remove high frequency noise and reaction rates were calculated as the first time derivative. Lines and shaded areas represent the average and standard deviation of the four individual reaction rates for each experimental condition. (**C**) Magnification of the reaction rate minima. For rapid 0 g–1 g-transition (Run1, blue line), the reaction rate reaches a minimum 3 min after the return to 1 g (blue arrow), followed by an increase. For stepwise increase of gravitational force (Run2, red line), the minimum of the reaction rate was detected between 0.3 and 0.5 g (red arrow), followed by an increase.

For comparison across experimental conditions, the raw signals were synchronized in time (setting the time of zymosan injection as t_0_) and normalized by their peak intensity thereby accounting for variations in the time of reaction initiation and PMT readings. The normalized signals (Fig. 6a) testify for the reproducibility within a given experimental condition. The average of the first derivative of the four independent PMT signals of Run1 revealed a minimum of the first derivative 3 min after the return to 1 g (blue arrow), followed by an increase which could indicate re-adaption to the 1 g gravitational environment (Fig. 6b,c). For stepwise increase of the gravitational force (Run2), the minimum of the first derivative was detected between 0.3 and 0.5 g (red arrow), followed by an increase, which may suggest a threshold of sensitivity to gravity between 0.3–0.5 g (Fig. 6b,c). Therefore, all four independent experiments of Run2 exhibited an immediate inhibitory effect of microgravity on the oxidative burst reaction, followed by a full adaptation to the new gravitational environment in less than one minute (Fig. 4). Furthermore, we detected rapid re-adaption to the 1 g environment and indications for a gravitational threshold of the oxidative burst reaction between 0.3 g and 0.5 g (Fig. 6). In contrast, Run1 exhibited no detectable difference to the ground reference experiments during the initial phases of the burst reaction including the step-wise g-level increase (Fig. 6a). The comparison of the data points of the averaged curves of all four experimental runs showed that for the half time up, the maximum of the curves and the half time down values, Run1 is similar to the technical ground reference experiments GRE1 and GRE2, which demonstrates that stepwise reduction of the gravitational force in 0.1 g increments – compared to the 1.0 g increment of Run2 - was not transduced into a detectable functional cellular reaction to altered gravity (Fig. 6a and Table 1). However, Run2 reaches the designated data points up to 9 minutes earlier indicating an enhanced burst reaction (Fig. 6b and Table 1). Differences may be explained by super-fast adaptation processes, which are probably below the low limit of detection for changes of 0.1 g (Run1), but detectable for changes of 1.0 g (Run2).

**Table 1:** Temporal characteristics of the oxidative burst reaction measured in the different experimental conditions, expressed as mean ± standard deviation over the four corresponding PMTs. For GRE1, PMT3 was a clear outlier and removed from the analysis.

Experiment	Half time up (min)	Time of maximum (min)	Half time down (min)
GRE 1 (n = 3)	7.29 ± 0.69	23.16 ± 0.50	54.62 ± 0.83
GRE 2 (n = 4)	9.67 ± 0.33	25.67 ± 1.49	54.15 ± 0.74
Run 1 (n = 4)	7.68 ± 0.61	23.97 ± 1.77	54.31 ± 1.99
Run 2 (n = 4)	5.48 ± 0.19	17.14 ± 1.04	51.33 ± 5.49

Time of maximum refers to the time when the curve reached its maximum value. The half time up and half time down refer to the times when the signal reached 50% of its maximum value during the initial amplification and subsequent decaying phases, respectively. For comparison across experimental conditions, these characteristic times are expressed as time since zymosan injection.

## Discussion

From the first clinical observations of a disturbed immune system in spaceflight^12, 13^, through the discovery of cellular sensitivity to gravity in lymphocytes^14^ to animal experiments^15, 16^ and detailed molecular studies^17, 18^, there is mounting evidence that gravity is obviously required for the normal functioning of immune cells^19^. This evidence also implies that immune dysregulation has the potential to be a serious clinical risk for exploration class lunar or Mars missions^20^. However, most of our knowledge about the effects of spaceflight on the immune system resulted from the analysis of samples subjected to short spaceflights or ground-based simulations^17^ and cellular experiments performed so far were endpoint measurements, analyzed on ground. The exact cause of the observed changes to the immune system from spaceflight environment has remained undiscovered^21^. In our experiment, we were able to provide for the first time, direct evidence of cellular sensitivity to gravity through real-time on orbit measurements using an experiment system of NR8383 rat macrophage cells, in which all factors except gravity were constant. Our previous results of a rapid decline of the oxidative burst reaction during a parabolic flight experiment^10^ could be confirmed by this independent ISS experiment, with completely different hardware, logistics, experiment process and cell culture conditions, indicating a very robust and reproducible finding. Importantly, in all 2D clinostat ground experiments conducted with exactly the same experiment system and in preparation for the ISS experiment TRIPLE LUX A, no adaptation was detected^10, 22^. Therefore, our studies provided not only direct evidence of sensitivity and rapid adaptation of mammalian cells to a microgravity environment, but also clear indications that adaption mechanisms react directly in response to microgravity, because they were not activated in a simulated microgravity environment of sedimentation-free and vector-averaged gravity.

The molecular mechanisms with which oxidative burst reacts and adapts to a new gravitational environment are yet unknown. Discussions of whether an *in vitro* single cell or a cell population can sense changes in the gravitational field are very controversial and theoretical considerations suggest that the forces involved are too small to trigger any cellular response to the changed environment^5, 23^. In spite of these theories, experimental data indicate that several types of cultured cells are sensitive to gravity^24, 25^, pointing to the cytoskeleton as a potential initial gravity sensor^26^. In our study we demonstrated direct evidence of a cellular response to microgravity. Additionally, the rapid reaction and adaptation of oxidative burst reaction suggests a direct effect at the level of the membrane-bound NADPH oxidase complex, which is closely associated with cytoskeletal dynamics^27, 28^ and linked to areas where tension and compression sensitivity are located^2^. RhoGTPases are interesting candidates to explain the structural cellular changes in microgravity^29^. As demonstrated in bone marrow polymorphonuclear leukocytes, RhoA activity not only responds within seconds upon stimulation, but is also associated with NADPH oxidase activation and oxidative burst reaction in time frames less than one minute^30^. In a previous study, repression of RhoA activation in bovine brain microvascular endothelial cells was observed after 72 h simulated microgravity (3D clinorotation), following down regulation of Rho-activating LARG (leukemia-associated Rho guanine nucleotide exchange factor) expression^31^. Importantly, p67phox, an essential structural component of the NADPH oxidase complex, is a target of Rac 1^32^, which is crucial for microgravity-induced alterations of the cytoskeleton and focal adhesions, demonstrated in MG-63 osteoblast-like cells silenced for RhoGTPases during the Foton M3 satellite mission^33^. Therefore, decrease of RhoA and increase of Rac1 activity could explain the rapid adaptation of the oxidative burst reaction in microgravity. Such dynamic reactions should be investigated in live-imaging studies as soon as this technology becomes operational for on-orbit experiments at ISS.

Numerous studies have reported that an altered gravitational environment or spaceflight have various and complex effects on cells and organisms, including cells of the monocyte-macrophage system^34–36^. Cells of the monocyte-macrophage system in microgravity demonstrated disturbed cytokine release^37–39^, reduced oxidative burst^10, 36^, alteration of the cytoskeleton^40^, reduction in their locomotion ability^41^, significant changes in gene expression associated with macrophageal differentiation^42^ and activation of syk signal pathway^43^ in microgravity. Because of the demonstrated direct evidence of ultra-fast adaptation mechanisms, results from previous *in vitro* and *in vivo* experiments in microgravity and in space^14–18, 24, 25, 34–36^ could represent not only and necessarily direct and primary effects of microgravity, but also molecular hallmarks of adaption processes, which may be initiated as fast as a few seconds after the onset of microgravity.

These adaptations appear to include very complex changes of cellular and molecular parameters. Due to the fact that gravity has been constant throughout the history of Earth and evolution of life^1^, no pre-set adaptation program or genetic memory of life responding to gravitational force changes can be expected. Cellular response to altered gravity may be less organized than other adaptation processes, yet many of the so far investigated terrestrial organisms are able to perceive gravitational forces in the range of 10^−3^ g. This happens in spite of the Earth’s acceleration of 1 g, which has been constantly present over millions of years, an enigma named the “gravi-paradox”^2^.

The weakening of the immune system in microgravity conditions triggered a lot of concern about human health during exploration class missions^17^. While there is much knowledge about the immune status that immediately follows the conclusion of a spaceflight, the understanding on immunity during spaceflight is still limited^19^. Research on the ISS may finally determine the immune status during long-duration spaceflight, and the successful closure of this clinical risk and, if required, by using the appropriate countermeasures^20^. Humans are ready to accept great risks in order to venture where no one has gone before, but there is a need for sufficient and sound biological information in order to support prolonged space habitation^17^. Our results suggest that mammalian cells may be equipped with an extraordinary fast and efficient adaptation potential when challenged with low gravitational environments. Interestingly, maize roots demonstrated the opposite effect, an enhanced oxidative burst in microgravity, which could be directly linked to the role of ROS as a messenger during the gravi-tropic response in plants^44^.

Like other environmental factors, the gravitational force may determine the boundaries for life^1^, where exploring the physical limits of organismic viability is crucial in the search for life in extraterrestrial habitats by narrowing down possible targets to search^45^. Our results imply that mammalian cells are equipped with a surprisingly ultra-fast and efficient adaptation potential to low gravity and that therefore key cellular functions of multicellular life could adapt to and exist in a low gravity environment. Since RhoGTPases are interesting candidates to explain the rapid adaptation of the oxidative burst reaction in microgravity, these molecules should be considered as interesting targets for countermeasures against microgravity-related cellular dysfunctions. However, dynamic monitoring of molecular activities in real microgravity is of crucial importance, and appropriate live imaging experiments on board of the International Space Station will be the key to understand the molecular dynamics of cellular adaptation to microgravity in the future.

## Methods

The TRIPLE LUX A experiment was performed in the BIOLAB laboratory of the European laboratory module Columbus on the ISS. The production of reactive oxygen species (ROS) in NR8383 rat alveolar macrophages after stimulation with the yeast cell wall component zymosan under altered gravity conditions was measured by the luminol reaction using photomultiplier detector systems^10, 11, 46^.

### NR8383 macrophage cell culture

NR8383 macrophageal cells of an early passage were thawed and cultivated in cell culture medium (HamsF12 (Biochrom Cat. No. FG0815) supplemented with 10% FCS (Fetal Calf Serum) filtered through a 0.2 µm sterile filter (Biochrom Cat. No. S0615, Lot No. 0323 W) and 0.1% 2-Mercaptoethanol (50 mM) filtered through a 0.2 µm sterile filter (Invitrogen/Gibco Cat. No. 31350–010)). Cells were seeded with a concentration of 0.2 × 10^6^ cells/ml, fed after 24 h with an equal volume of cell culture medium and after further 24 h split and re-seeded with a concentration of 0.2 × 10^6^ cells/ml. This process was repeated until sufficient cell material for cell freezing was obtained (see also Supplemental material).

### Evaluation of cellular burst capacity and cell freezing

Only cells that show a burst signal exceeding 200’000 relative light units (RLU) before freezing passed the quality control and were accepted to be frozen in SCBs (in cell culture medium containing 5% DMSO at a concentration of 2.7 × 10^6^ cells/ml) and used for the actual space and ground reference experiments. Aliquots of the frozen NR8383 cells were thawed and used for quality control experiments.

### Space flight and ground reference experiment hardware

The main hardware consisted of: (i) Stock Culture Bags (SCB), containing NR8383 rat macrophageal cells, (ii) Extra Liquid Bags (ELB), containing the chemicals, (iii) the Integrated Advanced Experiment Container (I-AEC) containing luminescence detector systems and (iv) the Handling Mechanism Plate Unit (HMPU) containing different compartments for liquid storage and transfer and experiment execution (Figs 1 and 2). Prior to the mission, the hardware was prepared, sterilized with ethylene oxide and degassed for 59 days. After this, the hardware was ready to be used for the pre-filling procedures at the University of Zurich and Space Life Science Labs at the Kennedy Space Center (KSC). Figure 2 illustrates the volumes and concentrations at each step of the experimental timeline. Briefly: NR8383 cells were transferred into SCBs and chemicals (luminol, zymosan, HRP) were transferred into ELBs. SCBs and ELBs were stored and transported at −80 °C. The HMPUs were pre-filled with buffer solutions and cell culture medium at the KSC and stored at 4 °C until launch (see also Supplemental material).

### Launch

The TRIPLE LUX A experiment was launched with Space X CRS-6 (Falcon 9 v1.1/Dragon Spacecraft) on 14 April 2015, 20:10:41 UTC from Cape Canaveral SLC-40, berthing date 17 April 2015, 13:29 UTC. NR8383 cells, chemicals and hardware were stored after docking at the designated temperatures on board the ISS until the start of the experiment.

ESA Astronaut Samantha Cristoforetti conducted both Run1 of TRIPLE LUX A on 29 April 2015 and Run2 on 6 May 2015. GRE1 and GRE2 were conducted on 19 May 2015 and 21 May 2015. Both GRE1 and GRE2 again included four independent experiments each.

### Experiment sequence

The experiment on board the ISS started with pre-warming the BIOLAB Rack to 37 °C. Thereafter, the pre-filled HMPU was integrated into the BIOLAB centrifuge. The ELBs were thawed and mixed manually by the astronaut and implemented into the Automatic Ambient Stowage-Insert (AAS-I). Subsequently, the NR8383 cells in the SCB were thawed and mixed by the astronaut and integrated into the designated space in the HMPU (see Fig. 2). The following experiment sequence inside the BIOLAB Rack proceeded automatically. Liquids were transferred by the automated handling mechanism (HM) from the AAS-I into IFB1 and IFB2 and from the SCB into MB1-4. The HMPU containing cells and chemicals was centrifuged at 1 g at 37 °C for 3 h for cell recovery. The measurement started by recording of the background signal, followed by pumping of liquids from IFB1 and IFB2 into MB1-4 to start the oxidative burst reaction. After injection of the fluids from IFB1 and IFB2, the concentrations in MB1-4 were 0.44 × 10^6^ NR8383 cells per ml, 1.7 mM luminol, 1.1 mg/ml zymosan and 55.85 U/ml HRP. A baseline signal for the oxidative burst reaction under 1 g conditions was recorded for 5 min before the centrifuge regimes specific for Run1 and Run2 were followed (see Fig. 3). The centrifuge regimes consisted of internal 0 g and 1 g controls and step-wise decrease (Run1) or increase (Run2) of gravity levels (Fig. 3a). Each one of these two runs included four independent experiments conducted in parallel. Real-time luminescence data was recorded in four independent parallel experiments and sent down to the Microgravity User Support Center (MUSC, German Aerospace Center) where the oxidative burst signal of the cells could be followed live. After the flight, the data was analyzed and processed when necessary. The ground reference experiments (GRE1 and GRE2) were conducted in the Biolab engineering model (EM) following the same procedure as the flight experiments except that there was no centrifugation included. Instead, the cells recovered for 3 h under Earth 1 g conditions at 37 °C.

### Experiment development, optimization and validation

We developed, tested, optimized, validated and established all experimental conditions, margins, timelines, operational and logistics procedures. These processes took place in labs in Switzerland (University of Zurich), in Germany (German Aerospace Center) and in the United States (Space Life Science Laboratory at Kennedy Space Center). Of particular interest are the two following points (for additional testing and optimization see supplement):

- *Cell viability during on-orbit experiments*: Although cell viability measurements were not suitable for standardization of the oxidative burst reaction, we monitored cell viability according to the mission and on-orbit scenario including freezing/thawing in 43 timeline experiments (following the exact times as defined for the on-orbit operations) with measurements in 131 individual samples and an average cell viability of 80.69 +/− 11.02% at the end of the timeline. Viability of all cells used in the on-orbit experiment were measured in a similar timeline on ground and revealed always >80% viability.
- *Verification of linear flow and maximal acceleration*: Stirring is necessary to ensure a homogeneous distribution of cells, nutrients, chemicals and gas. Since it has to be excluded that mechanical stress induced by stirring disturbs the microgravity experiment, the linear fluid flow (Reynolds number <125) and maximal stirring-induced acceleration (0.00637 g for 60 rpm and of 0.02548 g for 120 rpm) has been calculated for the worst-case scenario (document IEC-AN-163021-3002-EPC). It could be verified that mechanical forces induced by slight stirring did not interfere neither with the on-orbit nor with the ground experiments.

### Data Analysis

**Figure 7:**
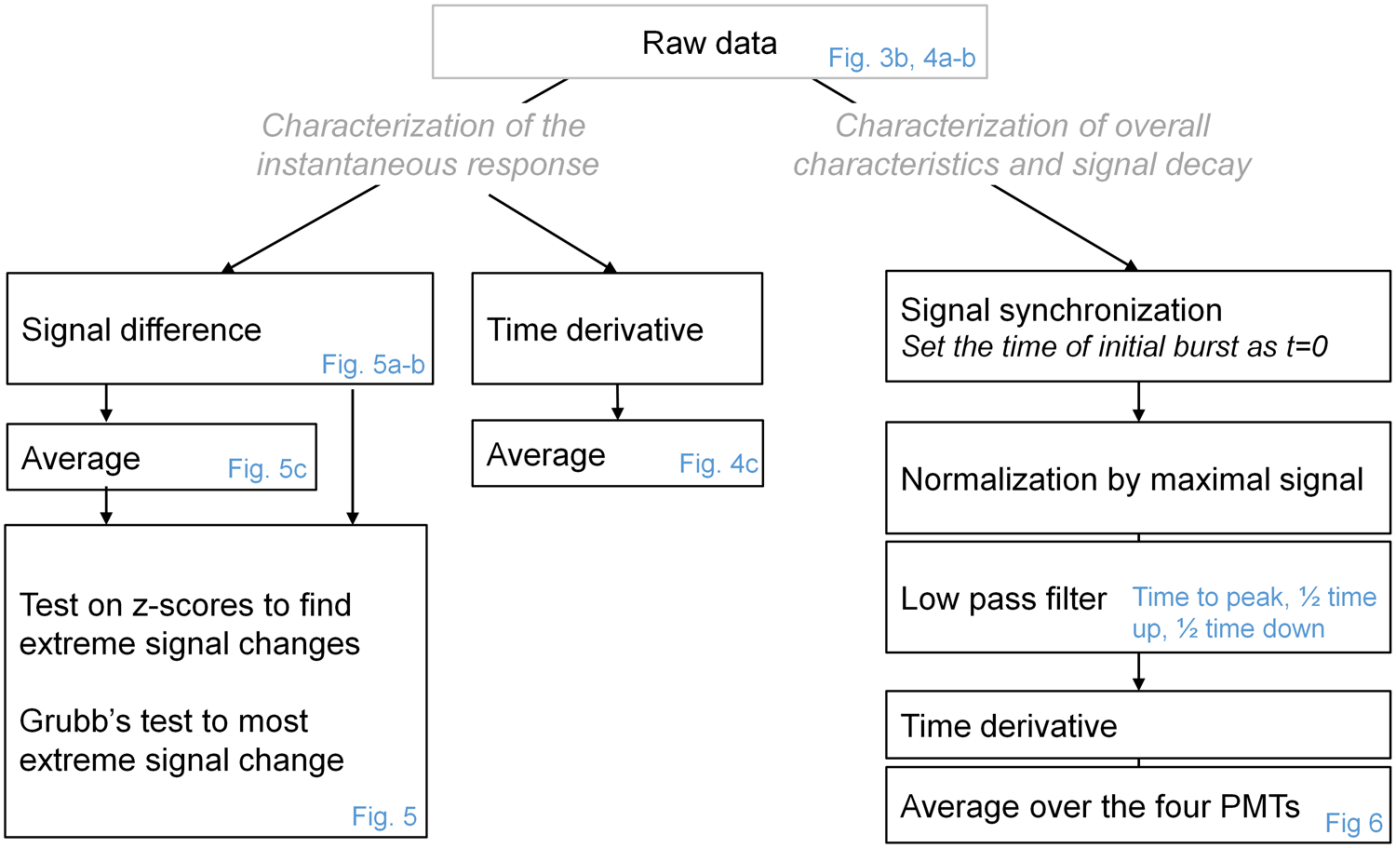
Data analysis workflow. Blue labels depict the main metrics or figures derived from the different analysis steps. For GRE1, PMT3 was a clear outlier and removed from the analysis.

We could observe in the raw signals from Run2 orbit experiment (see Fig. 4) that a burst signal decrease happened at the transition time when gravity changed from 1 g to 0 g. This observation was confirmed when further investigating the signal changes in more detail. The data processing workflow is illustrated in Fig. 7.

One analysis was done with the raw data without any signal filtering or normalization (left branch in Fig. 7). Signal changes were quantified on the raw signal as either the signal time derivative (Fig. 4c) or the signal difference between consecutive time points (Fig. 5). For all four PMTs we observed an extreme signal decrease (large negative signal difference and derivative) Heiko Mantzsch and Luca Briganti. We are very thankful for the outstanding upon onset of microgravity. For three out of the four PMTs, the strongest change was found to be the signal decrease upon onset of microgravity. Despite the weaker change in PMT1, the average of the raw signal differences across all four PMTs showed the strongest signal change of all 28’199 calculated signal differences at the time when the g-level changed from 1 g to 0 g (see blue lines in Fig. 5C). A Grubbs’ test was performed to quantify the significance of the observed effect, revealing high evidence for all four individual signal differences as well as for their average (p-value ≪ 0.0001). This test was developed by Grubbs^48^ to investigate if at “some time during the experiment something possibly happened to cause an extraneous variation on the high side or on the low side”^47^ and is also nowadays routinely used in regression model checking procedures (i.e. to find outliers in Cook’s d values or standardized residuals). In addition, we determined for each signal difference if it shows a significant deviation from a normal variation around zero (quantified by a test on z-scores given by the differences between each value and the mean divided by the standard deviation) – red points mark significant signal jumps on a significance level of alpha = 10^−10^. This Statistical analysis was performed using R version 3.1.0 [R Core Team. R: A Language and Environment for Statistical Computing. Vienna, Austria: R Foundation for Statistical Computing; 2014. http://www.R-project.org/].

To get a better visualization of the changes occurring during the signal decay upon restoration of gravity, we performed a second data analysis involving some data processing (see right branch in Fig. 7). In the raw data of all experiments we can easily identify the time of zymosan injection reflecting the initiation of the reaction. The PMT recordings (Fig. 3B) were thus first synchronized, setting the time of zymosan injection as reference time for all signals. Next, signal intensities across PMTs display significant amplitude variations, which prevent direct comparison across PMTs and experimental conditions. Furthermore, while the downward spike of Run2 can clearly be seen on the temporal derivative or signal difference of the raw signal, noise prevents any clear interpretation of the derivatives for any other parts of the signal. Accordingly, the following signal processing steps were taken for the analysis of the decaying section and overall temporal characteristics: (i) Each PMT signal was first normalized by its peak amplitude; (ii) The normalized signals were filtered using an alternating direction zero-phase filter with kernel size 50, to remove high frequency components without introducing phase distortion; (iii) The time to peak, half time up and down (defined as the time when the signal amplitude reached 50% of its peak value) and time derivatives were computed for each filtered signal. When relevant, data were averaged over the 4 PMTs corresponding to one experimental condition and reported as mean ± one standard deviation. For GRE1, PMT3 was a clear outlier and removed from the analysis. This signal processing was performed in Matlab 2015 (The MathWorks Inc., Natick, MA, USA).

## Electronic supplementary material

Supplementary Information
