# Supplementary material for: Rapid adaptation to microgravity in mammalian macrophage cells
Source: Sci Rep. 2017 Feb 27;7:43. doi: 10.1038/s41598-017-00119-6 (PMC5427920; doi:10.1038/s41598-017-00119-6)
Supplement: Supplementary file 1 — Supplementary Information [file 41598_2017_119_MOESM1_ESM.pdf]

Supplementary Material

Supplement to Material and Methods
of the International Space Station experiment TRIPLE LUX A

Rapid adaptation to microgravity in mammalian macrophage cells

Cora S. Thiel[1,4,5,*], Diane de Zélicourt[2], Svantje Tauber[1,4,5], Astrid Adrian[6], Markus Franz[6], Dana M. Simmet[1,4], Kathrin Schoppmann [1], Swantje Hauschild[1,4,5], Sonja Krammer[1], Miriam Christen[1], Gesine Bradacs[1], Katrin Paulsen[1], Susanne A. Wolf[1], Markus Braun[8], Jason Hatton[9], Vartan Kurtcuoglu[2,3], Stefanie Franke[7], Samuel Tanner[10], Samantha Cristoforetti[9], Beate Sick [11], Bertold Hock[12], Oliver Ullrich[1,3,4,5,*]

(1) Institute of Anatomy, Faculty of Medicine, University of Zurich, Winterthurerstrasse 190, 8057 Zurich, Switzerland
(2) Institute of Physiology, Faculty of Medicine, University of Zurich, Winterthurerstrasse 190, 8057 Zurich, Switzerland
(3) Zurich Center for Integrative Human Physiology (ZIHP), University of Zurich, Winterthurerstrasse 190, 8057 Zurich, Switzerland
(4) Department of Machine Design, Engineering Design and Product Development, Institute of Mechanical Engineering, Otto-von-Guericke-University Magdeburg, Universitätsplatz 2, 39106 Magdeburg, Germany
(5) Space Life Sciences Laboratory (SLSL), Kennedy Space Center, 505 Odyssey Way, Exploration Park, FL 32953, United States of America
(6) Airbus Defense and Space, GmbH, Claude-Dornier-Strasse, 88090 Immenstaad, Germany
(7) German Aerospace Center (DLR), Linder Hoehe, 51147 Cologne, Germany
(8) German Aerospace Center (DLR), Space Agency, Königswinterer Strasse 522-524, 53227 Bonn, Germany
(9) European Space Agency (ESA), Keplerlaan 1, 2201 AZ Noordwijk, Netherlands
(10) BIOTESC, CC Aerospace Biomedical Science and Technology, Lucerne School of Engineering and Architecture, Technikumstrasse 21, 6048 Horw, Switzerland
(11) Epidemiology, Biostatistics and Prevention Institute (EBPI), University of Zurich, Zurich, Switzerland
(12) Chair of Proteomics and Bioanalytics, TU München, Alte Akademie 14, 85354 Freising, Germany

Contents

1 Macrophageal production of reactive oxygen species as a measure for cell reactivity under altered gravity conditions 3

2 Pre-flight Optimization and Validation 4

3 Experimental preparation 6

3.1 Common preparation at the University of Zurich 6

3.1.1 NR8383 macrophageal cell culture 7

3.1.2 Hardware filling 7

3.1.3 Quality control 8

3.2 Transport of hardware to the launch site 9

3.3 Pre-Mission Schedule at the Space Life Sciences Laboratory (SLSL), KSC 9

3.3.1 Hardware pre-filling 10

3.3.2 Liquid transfers for Ground Reference and on orbit experiments 10

3.4 Sample handover to NASA and sample upload 11

4 International Space Station operations 11

4.1 Biological Experiment Laboratory (BIOLAB) 11

4.2 Launch 12

4.3 TPLX-A Experimental Sequence Overview 13

4.4 Detailed TPLX-A Experimental Sequence 13

4.5 BIOLAB centrifuge regime 14

4.6 In-flight measured parameters 14

4.7 Ground reference experiments 14

4.8 Management 15

5 Abbreviations 16

1 Macrophageal production of reactive oxygen species as a measure for cell reactivity under altered gravity conditions

The **TRIPLE LUX A (**TPLX-A) experiment was performed to elucidate the mechanisms underlying the impaired functions in immune cells observed during spaceflights. Normally, macrophages and vertebrate immune cells kill pathogens by phagocytosis followed by reactive oxygen burst to degrade the pathogen. The reactive oxygen species (O_2^-) is generated as a byproduct of oxidation of NADPH by the NADPH oxidase enzyme.

In the TPLX-A experiment the reactive oxygen burst was used as a quantitative measure of the phagocytic activity of macrophages under spaceflight conditions. Monocyte-macrophage cells (NR8383, ATCC# CRL-2192) derived from rat (*Rattus norvegicus*) were used as model organism. The burst capacity of the immune cells was analyzed by a chemiluminescense assay where O_2^- radicals convert luminol to 3-Aminophthalate, which results in the emission of light at 475nm (see figure S1). The luminol reaction was catalyzed by the enzyme horseradish peroxidase (HRP) resulting in an enhanced light emission. The yeast cell wall polysaccharide zymosan functioning as an analogue of bacteria was used to stimulate the macrophages and to initiate the reactive oxygen species (ROS) production.

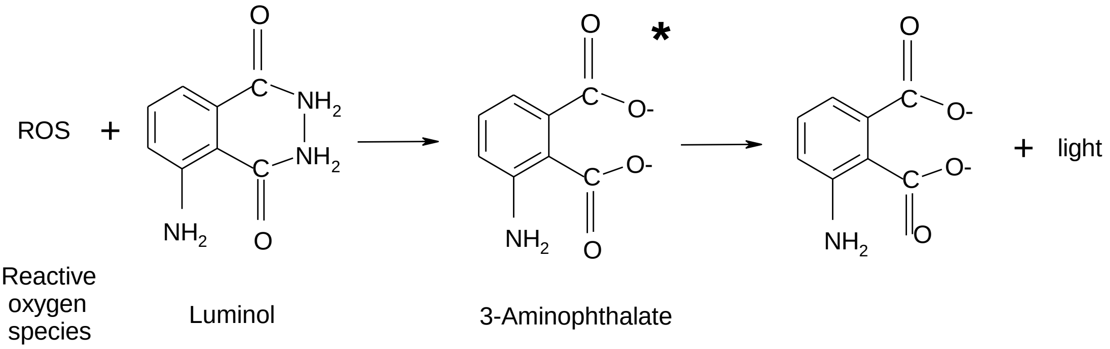

Figure S1: Luminol reaction

2 Pre-flight Optimization and Validation

Between March 2012 and June 2014, a large number of experiments were conducted to carefully optimize all experimental parameters for chemical stability, cell viability and burst signal intensity before launching the actual on orbit and ground reference experiments. Table S1 summarizes the parameters that were investigated and the optimal settings retained, while Table S2 depicts the optimized experimental timeline.

After the optimization of the experimental procedure, the actual experiment sequence had to be thoroughly tested in order to ensure a smooth process in space. This was done with incremental complexity in the three following test sequences.

1) Mini-Science Validation Test (mini-SVT): a pre-validation in a lab setting
2) Science Validation Test (SVT): validation in a flight-analog scenario, performed at MUSC, DLR (Cologne) in the BIOLAB engineering model from June 2 to June 6 2014
3) Experiment Sequence Test (EST): simulation of the whole flight scenario from the preparation of the experiment containers in the laboratory to the on-board workflow. Performed between October 24 and November 12, 2014 in Zurich (preparation) and at the MUSC in Cologne (orbit simulation)

Sections 3 and 4 detail the experimental protocols as performed for the reported on-orbit and ground reference experiments, after optimization and approval by the space agencies DLR and ESA.

Table S1: Summary of the pre-flight optimization and validation experiments

	Parameter & Objective	Optimum retained for subsequent experiments
**Bioassay for ROS detection**	Luminol based bioassay for detection of reactive oxigen species (ROS)	Final concentrations: 1.7mM luminol (Sigma Cat No. A4685), 1.1 mg/ml zymosan (Sigma Aldrich Cat No. Z4250) opsonized with donor horse serum (Biochrom), 55.85U/ml HRP (Merck Cat.No. 516531),0.44x10^6 NR8383 cells/ml
	Temperature sensitivity of the oxidative burst measurement	Oxidative burst reaction kinetics are very temperature sensitive. Temperature should be tightly controlled (37°C)
	Horseradish peroxidase (HRP) provider and concentration	Horseradish peroxidase from Merck (Cat No. 116216) at a working concentration of 55.85 U/ml
**Hardware and consummables**	Cell culture flasks	T175 blue cap flasks (BD Bioscience Falcon cat. no. 353112)
	96 well plates suitable for the plate reader Synergy2 (BioTek)	Greiner bio-one cat. no. 655061
	Cell toxicity of the BIOLAB hardware	No cell toxicity noted
	Sterilization method for hardware sensitive to ethanol, isopropanol and gamma sterilization	Sterilization with 100% ethylene oxide gas. No bacterial growth noted in any of the tests. Cell viability and functionality required 59 days degassing after sterilization.
	Cell damage induced by hardware needles during aspiration and injection	Handling with BIOLAB custom-made syringes was less detrimental than other commercial alternatives, but the following guidelines were still imposed to minimize damage:<br>- Minimize the number of transfer<br>- Lowest possible transfer velocity (≤ 0.2 ml/sec)<br>- Cell concentration of 2.7x10^6/ml
**Pre-freezing culture**	Cell culture media storage temperatures and times	Storage at 4°C up to 11 months yielded results comparable to positive control. Storage at ambient temerature or freezing at -20°C and thawing were unsatisfactory.
	NR8383 cell concentration for freezing	2.7x10^6/ml
	Correlation of the cell reactivity before and after freezing	The burst capacity of the cells should be > 200'000 RLU before freezing to yield reasonable signals after freezing
	Optimal freezing medium composition	Ham´s F12 medium (Biochrom) supplied with 10% fetal calf serum (Biochrom), 50µM 2-mercaptoethanol (Gibco), and 5% DMSO (Sigma)
	Fetal calf serum of US origin (as per US regulation)	Similar results for both (Sigma F2442; Lot No.: 14E332) and (Biochrom S0615, Lot No. 0323W)
	Titration of HEPES (used to buffer pH changes due to the lack of CO_2 control on board the ISS)	5mM
**After thawing**	Chemical stability after thawing	Stable for all tested times (up to 835min)
	Most efficient shaking procedure to homogenize cell suspension after thawing in micro-gravity	On orbit shaking sequence after thawing:<br>1) Circular motion for 30 seconds<br>2) Vigorous back and forth lateral shaking for 10 seconds
**Stirring**	Optimal stirring sequence for proper mixing of the cells and media	- Stirring at 60 or 120rpm for 1 min every hour during recovery<br>- Continuous stirring at 60rpm or 120rpm during measurement
	Estimation of the forces induced by stirring to ensure that they are lower than 1g	Maximum force in the measurement bag estimated to be 0.006g at 60rpm and 0.025g at 120rpm, i.e. well below the investigated 0.1g or 1g increments
**Storage**	Evaluation of maximum storage time at -80°C without quality loss	6 months for both NR8383 cells and chemicals
**Timeline**	Evaluation and optimization of all individual steps	Timeline shortening from 835 min to 415 min (see table S2)

Table S2: Optimized cell concentrations in the stock culture bags (SCBs) and measurement bags (MBs), temperatures and experiment timeline steps that were applied for both on orbit and on ground experiments.

**Cell concentration**	**Temperature**	**Time of measurement**
SCB<br>$2.7x10^6/ml$	25°C	T: 0min<br>Thawing
SCB<br>$2.7x10^6/ml$	37°C	T: 55min<br>Ambient to 37°C (for 180min)
MB<br>$0.44x10^6/ml$	37°C	T: 290min<br>Start measurement (for minimum 125min)
MB<br>$0.44x10^6/ml$	37°C	T: 415min<br>End of measurement

3 Experimental preparation

Preparation of the on orbit or ground reference experiments involves a large number of steps, all of which may introduce confounding effects in the end result if not carefully designed and monitored. The following sections briefly describe the procedures to equipe the reader with all knowledge required for the critical analysis of our results.

3.1 Common preparation at the University of Zurich

For all experiments, pre-freezing cell culture, chemical preparation and hardware sterilization were conducted (Figure S2) following the optimized protocols detailed above. The exact same protocols were followed irrespective of the samples' end-destination.

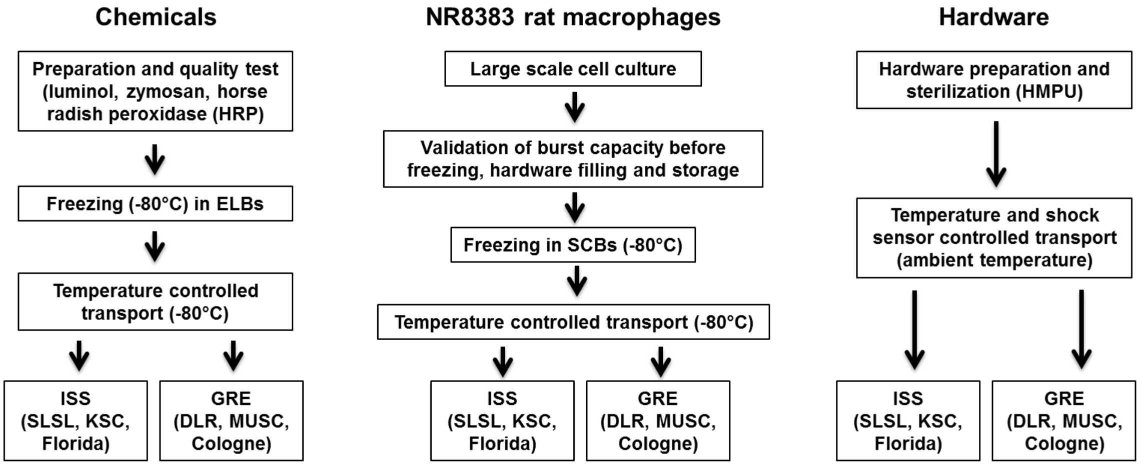

Figure S2: Overview of the experimental preparation sequence before transfer and handover to NASA and DLR for the on-orbit and ground-reference experiments, respectively. All preparation steps were conducted at the University of Zurich (Zurich, Switzerland) following identical protocols irrespective of the end destination.

3.1.1 *NR8383 macrophageal cell culture*

NR8383 macrophageal cells of an early passage were thawed and cultivated in cell culture medium (HamsF12 (Biochrom Cat.No. FG0815) supplemented with 10% FCS (Fetal Calf Serum) filtered through a 0.2 µm sterile filter (Biochrom Cat. No. S0615, Lot No. 0323W) and 0.1% 2-Mercaptoethanol (50mM) filtered through a 0.2 µm sterile filter (Invitrogen / Gibco Cat. No. 31350-010)) three weeks before the hardware filling. Cells were seeded with a concentration of $0.2x10^6$ cells/ml, fed after 24h with an equal volume of cell culture medium and split and re-seeded with a concentration of $0.2x10^6$ cells/ml after further 24h. At day 6 the cell culture was split into four parallel lines, which were cultivated in four different incubators and processed in two different cell culture laboratories. The best performing sub-line was chosen and the cell culture flasks were devided into two equal batches to fill six stock culture bags (SCBs) each. It was necessary to split the cell freezing into two batches, because the duration of the filling process is very critical and the burst capacity of cells is reduced highly if cells are incubated too long after detachment from the cell culture flask.

3.1.2 *Hardware filling*

On the day of hardware filling the freezing medium and chemicals for the extra liquid bags (ELBs), were degassed and stored at 4°C. The hardware filling was performed under a clean bench to maintain sterility of the samples. All hardware components were sterilized before via exposure to ethylene oxide gas and degassed for 59 days. All consumables and plasticware were sterile.

The hardware filling was split into two parts.

- All solutions that could be stored and transported at -80°C were filled into the sterilized hardware in the laboratories at the University of Zurich. This included the SCBs and ELBs.
- The liquids that needed to be stored and transported at 4°C, i.e. the cell culture medium, 0.9%NaCl and 20mM borate buffer, were freshly perpared and filled into the respective compartments of the Handling Mechanism Plate Unit (HMPU) (see Fig.2) in the laboratories at the Space Life Science Laboratories (SLSL) shortly before the handover of the hardware to the NASA launch team.

The SCBs were filled with 4.2ml (±0.2ml) of NR8383 cell solution ($2,7x10^6$/ml in freezing medium, see table S1). Air bubbles were removed from the SCBs. After visual inspection, the complete SCB was placed in the CoolCell (a cell freezing container to ensure standardized

controlled-rate -1°C/minute cell freezing in a -80°C freezer) and stored at 4°C until the finalization of the second SCB. Then the CoolCell with both SCBs, 10 further cryovials filled with cells in freezing medium, and placeholder cryovials filled only with freezing medium was placed at -80°C. After 24 hours one cryovial was removed from the CooCell, thawed and used for a timeline experiment to guarantee the quality of the cell batch frozen in the SCBs. In total 12 SCBs were frozen. Those SCBs with the best test results in a timeline experiment were stored until transport either to the launch site at KSC or to MUSC, DLR where the ground reference experiments were performed.

Three ELBs were prepared for each chemical (luminol, zymosan, HRP). Chemical concentrations and volumes are listed in Table S3. The degassed solutions were filled into the readily assembled ELB with needle and syringe through the septum. Air bubbles were removed. The ELBs were frozen at -20°C. During the freezing process, the ELBs filled with zymosan were turned every 10 min to minimize pelleting of zymosan particles. The ELBs were stored at -20°C until the transport to the launch site. During transportation and upload ELBs were stored between -80°C and -95°C.

Table S3:Chemical concentrations in ELBs and transferred volumes to internal filling bags (IFBs).

**ELBs (volume 3.2 ml)**	**Concentration**	**Transferred volume to IFBs**
ELB Zymosan	18.4 mg/ml	2.5 ml
ELB-HRP	10150 U/ml	0.23 ml
ELB-Luminol	25mM	2.5 ml

3.1.3 Quality control

The following steps were taken to monitor quality throughout the process:

- Burst capacity of the cells was analyzed in a plate reader once a week throughout the cultivation period.
- Two days before the hardware filling, a sample with $2.7x10^6$ cells/ml was frozen from each sub-line. The next day samples were thawed and a full timeline was performed to analyze the burst capacity of the cells after thawing. All four sub-lines performed equally well.

- An aliquot of each of the four sub-lines of NR8383 cells was analyzed in a plate reader assay for their burst capacity. All four sub-lines fulfilled the burst before freezing threshold of minimum 200000 RLU. The sub-line with the highest counts (more than 2 mio RLU) and the best results during the beforehand tested timeline after freezing and thawing was chosen.
- For the chemicals, a functionality test was performed by using an aliquot for a plate reader assay together with an approved set of chemicals as a positive control.
- 24 hours after filling, one cryovial was removed from the CoolCell, thawed and used for a timeline experiment to guarantee the quality of the cell batch frozen in the SCBs.

3.2 Transport of hardware to the launch site

In total eight SCBs and nine ELBs were transported. The samples were transported to the Space Life Sciences Laboratories (SLSL) in Orlando, Florida, under strict temperature control in two portable ultra low freezers (Nanolytik) at -80°C. The freezers were connected to external battery packs and power supply was provided on board the aircraft and during custom clearance. ELBs were transported on dry ice. During the transport of more than 30 hours the temperature was permanently controlled and the dry ice was re-filled twice. After arrival of the samples at the SLSL the SCBs and ELBs were immediately stored at -80°C.

3.3 Pre-Mission Schedule at the Space Life Sciences Laboratory (SLSL), KSC

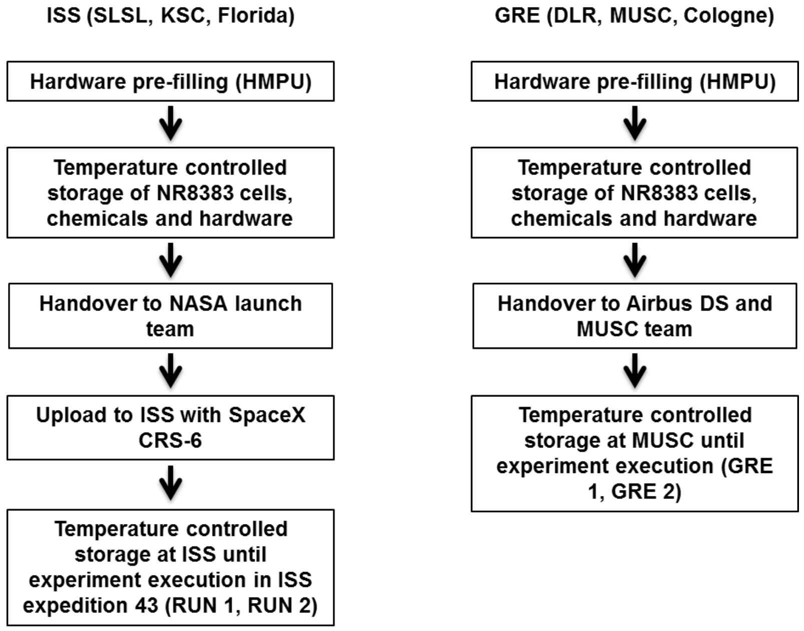

Figure S3: Pre-mission schedule for the on-orbit ISS experiments (NASA Space Center, Florida, USA) and ground reference experiments (DLR, Cologne, Germany).

3.3.1 Hardware pre-filling

Liquids were degassed under reduced pressure for 2 hours and stored for maximum 24h at 4°C until filling of the hardware.

Four different HMPUs were prepared for the on orbit experiment. Finally, two HMPUs were chosen to be uploaded to the ISS. The remaining two HMPUs were rinsed and shipped back to the USOC (DLR, Cologne) for the ground reference experiments.

The internal filling bags (IFBs) 1 and 2, measurement bags (MB) 1-4 and connecting tubings were carefully rinsed with cell culture medium under sterile conditions. The compartments were then filled as follows (see also Tables S4-5):

- *IFB1:* 1.5 ml of degassed borate buffer (20mM).
- *IFB2:* 2.27 ml of degassed NaCl solution (0.9%).
- *MB:* 2.4 ml of degassed cell culture medium containing all additives.

Air bubbles were removed at best. The HMPUs were stored at 4°C until handover to the NASA team. 24 and 48 hours after filling the single compartments were visually checked for the appearance of new air bubbles, which were removed with needle and syringe under sterile conditions.

3.3.2 Liquid transfers for Ground Reference and on orbit experiments

As soon as the SCB and the ELBs were inserted into the BIOLAB rack, the transfer of liquids was performed automatically. First luminol, HRP and zymosan were transferred from the ELBs into the respective IFBs with the Handling Mechanism (HM) using the Biolab syringes. Then the NR8383 cells were transferred from the SCB to the MB1-4. The velocity of transfer was <0.02 ml/sec.

Table S4: Chemical concentrations in IFB1and IFB2 and transferred volumes to MBs.

**IFB (total volume)**	**Composition**	**Concentration in IFB**	**Transferred volume to each MBs**
IFB1 (4ml)	2.5ml luminol (25mM)<br>1.5ml borate buffer (20mM)	15.625 mM	0.5 ml
IFB2 (5ml)	2.5ml zymosan (18.4mg/ml)<br>0.23ml HRP (10150U/ml)<br>2.27ml NaCl (0.9%)	9.2mg/ml<br><br>466.88U/ml	0.55ml

Table S5: Final chemical concentrations in MB1-4 and transferred volumes from IFBs to MBs.

**MB1-4**<br>**4.6 ml**	**Volume**	**Final conc.**
Pre-filled cc medium	2.4ml	-
Syringe transferred NR8383 cells from SCB ($2.7x10^6$ cells/ml)	0.75ml	$0.44x10^6$cells/ml
Pumped liquids from IFB1 (luminol)	0.5ml	1.7mM
Pumped cc medium pre-filled in tubings (IFB1)	0.2ml	-
Pumped liquids from IFB2<br>(Zymosan)<br><br>(HRP)	0.55ml	 1.1mg/ml<br><br>55.85U/ml
pumped cc medium pre-filled in tubings (IFB2)	0.2ml	-

3.4 Sample handover to NASA and sample upload

Samples, chemicals and pre-filled hardware were stored at the dedicated temperatures until the handover to the NASA launch/cold stowage team. During the upload with the Dragon space capsule (SpaceX CRS-6) the SCBs and ELBs were stored in an ultra low freezer at -95°C while the HMPU was stored with double cold bags at 4°C. On board the ISS the SCBs were stored in the Minus Eighty Laboratory Freezer (MELFI), the ELBs were stored in the first temperature controlled unit (TCU) of Biolab at -20°C and the HMPU was stored in the second TCU of Biolab at 4°C until the execution of the experiment. Samples and reagents could be stored for max. 6 months from the time of freezing.

4 International Space Station operations

4.1 Biological Experiment Laboratory (BIOLAB)

BIOLAB is the biological experiment facility in the Columbus module on board the International Space Station (ISS). It was launched and installed in the Columbus laboratory in February 2008 and is operational since then. This single-rack (International Standard Payload Rack - ISPR) payload was designed to perform life science experiments on e.g. microorganisms, animal and plant cells, small plants, tissue cultures and small invertebrates. The research supported by this experiment facility should help to elucidate the effects of microgravity or altered gravity and space radiation on single cells and multi-cellular organisms. The BIOLAB rack consists of an automated and a manual unit where the ISS crew can

participate in an experimental procedure. The automated core unit can either run autonomously via a loaded program or it can be operated by telecommanding from the ground control center.

Inside the Biolab ISPR the temperature, atmosphere, humidity and light intensity can be controlled. It houses various facilities that can be used. The automated section includes an incubator, two centrifuges, a robotic handling mechanism (HM), a microscope, automatic temperature controlled stowage (ATCS), and an automated ambient stowage (AAS). The experiment preparation unit (EPU), the BiolabGloveBox (BGB) and two temperature control units (TCUs) for storage of experiment containers (ECs) and samples belong to the manual unit. The advanced experiment containers (AECs) were designed with supplementary functions like video recordings or other integrated specialized measurement units. Inside the incubator the temperature can be controlled between 18.0 °C and 40.0 °C (+/- 0.5 °C). The centrifuges are placed inside the incubator and can provide gravity levels between 0.001 g up to 2 g with 0.001 g steps. The HM represents the interface between the AECs and the instruments used for measurements and can be used to tranfer samples from one compartment to another. The BioGloveBox allows the astronauts to safely manipulate the experiment hardware. Its control system allows to regulate the temperature between 21 °C and 38 °C (+/- 2°C). The temperatures in the two TCUs can be controlled between -20 °C and +10 °C (+/- 1 °C). Besides the two TCUs there is a possibility for sample storage at -80°C in the Station´s Minus Eighty Laboratory Freezer (MELFI). Samples and hardware units (e.g. AEC) that have been frozen are thawed in the BioGloveBox and are subsequently integrated into the automated BIOLAB section where the automated sample handling takes place. Usually, a ground reference experiment is performed in parallel or shortly after the experiment on board the ISS has been executed.

4.2 Launch

Launch of the TPLX-A experiment was on 14 April 2015, 20:10:41 UTC with a Space X CRS-6 Falcon 9 v1.1 from Cape Canaveral SLC-40. Berthing was on 17 April 2015, 13:29 UTC, at the ISS module Harmony using the nadir mechanism. The TPLX-A experiment was performed by Expedition 43, which commenced on 11 March 2015 with the undocking of Soyuz TMA-14M and ended with the departure of Soyuz TMA-15M on 11 June 2015. The experiment runs (Run1 and Run2) were conducted on 29th April and 6th May 2015, respectively, by ESA Astronaut Samantha Cristoforetti.

4.3 TPLX-A Experimental Sequence Overview

The experiment was composed of two consecutive runs performed within the same advanced experiment container (AEC). The two runs were performed in an interval of 7 days (tested margin: 6 month).

Operating steps:

1.) Thaw up reagents in ELBs (Peroxidase, Luminol, Zymosan). Transfer reagents by HM: Luminol from ELB to IFB1, Peroxidase and Zymosan from ELB to IFB2,
2.) Thaw stock cell cultures in the SCB (stock culture bags),
3.) Inject stock culture (NR8383 macrophage cells) directly from SCB to MB1-4 (measurement bags) prefilled with cell culture medium,
4.) Incubation at 1g and 37°C for 3h,
5.) Add Luminol, Peroxidase and Zymosan by internal pumping from IFB1 and IFB2 to MB1-4 to start the bioluminescence assay. Luminol is transferred first and the baseline signal is recorded for 10 min. Then, Peroxidase and Zymosan are pumped and the measurement of luminescence starts at 1g for 5 min, The exact volumes for each chemical transfer can be found in tables S4 and S5.
6.) Start of the centrifuge regime 1, measurement of luminescence from the culture during the BIOLAB centrifuge regime.

The second flight was performed one week after the first one following the same protocol with the centrifuge set at regime 2.

4.4 Detailed TPLX-A Experimental Sequence

The HMPU, the hardware unit where cells and chemical dilutions were performed and where the measurement took place, was integrated into the experiment container (EC) and installed on the rotor of the BIOLAB centrifuge in the incubator. The incubator was set to 37°C. Then, for each experiment, ELBs and SCB were thawed, and mixed and the SCB was integrated into the HMPU, while the ELBs were integrated into the Automatic Ambient Stowage (AAS) insert. Via ground control commanding from the user support center in Cologne, Germany (MUSC, DLR) the experiment program was started, supervised and controlled. Firstly, the HM took a BIOLAB-syringe out of the AAS insert, went to the ELB filled with luminol, aspirated the liquid and injected it into IFB1 of the HMPU. The syringe was put back in place, a new syringe was taken and the HRP mixture was aspirated from the second ELB and injected into IFB2 of the HMPU. The syringe was put back into the AAS. A clean syringe was taken by the HM and the zymosan solution was transferred from the ELB into IFB2. Then, with a new syringe, the

cells were aspirated from the SCB and injected into MB 1-4 of the HMPU one after the other. A 3 hours adaptation time at 37°C started where the HMPU on the rotor was centrifuged with 1g to simulate the normal gravitational acceleration of 9,81 m/s^2. After this adaptation, first luminol and then the zymosan/HRP solution were pumped into the MBs to start the oxidative burst measurement at 37°C accompanied by the appropriate centrifugation regime. Three weeks after the experiments on board the ISS, two ground reference experiments (GRE) were performed in the BIOLAB engineering model at the USOC in Cologne (MUSC, DLR).

4.5 BIOLAB centrifuge regime

On board the ISS two consecutive experimental runs for the TPLX-A project were foreseen to be executed within a timespan of maximum 4 weeks (Figure S11). For both experiments an initial adaptation phase of the cells for 3 hours at 1g was planned. After injection of luminol and zymosan/HRP, a baseline measurement was recorded for 5 min. Then, for Run1 the rotor speed was decreased every 2.75 min by 0.1g (10 steps) reaching finally 0 g. With this phase of the centrifuge regime we intended to identify a possible 1g to 0g threshold. The centrifuge stayed switched off for 15 min to analyze adaptation effects to microgravity. The centrifuge was started again at 1g to investigate the 1g re-adaptation potential of the cells. During on orbit run 2, the centrifuge was completely switched off after the 3 hours adaptation phase at 1g and the cell adaptation for the 1g - 0g transition was measured for 15 min before the centrifuge was re-started and a g-profile with stepwise increase of 0.1g steps (10 steps, 2.75 min each step) was run from 0g to 1g to investigate 0g - 1g thresholds and/or a 1g re-adaptation.

4.6 In-flight measured parameters

The chemiluminescence associated with the phagocytosis of the zymosan particles by the macrophages was measured for approximately 500 minutes. The luminescence (light emission at 450nm +/- 30nm) produced by the cells was recorded with a minimum sampling rate of 0.1Hz by the Photo Multiplier Tubes (PMTs) inside the ECs and the measured data was directly downlinked via telemetry and saved by the USOC (MUSC, DLR, Cologne).

4.7 Ground reference experiments

Cells and reagents from the same batch as the in-flight samples, were stored in the laboratory at the University of Zurich and kept at -80°C and -20°C respectively until the execution of the ground reference experiments (GRE). Cells, reagents and all necessary laboratory equipment were transported at the designated temperatures to the USOC (MUSC, DLR) in Cologne

Germany. Three weeks after the second experimental run on orbit, the two ground reference experiments (GRE1 and GRE2) were performed on 19.05.2015 and 21.05.2015. The experiments were performed inside the BIOLAB engineering model onsite the premises of MUSC. The same actual flight sequence was performed for all steps of the timeline.

4.8 Management

Many different parties with different expertises were involved in the planning and execution of the TPLX-A experiment. AIRBUS DS designed and constructed the hardware and was responsible for the hardware handling during the pre-mission tests as well as during the flight experiment preparations. The science team was responsible for the concept of the complete experiment including the design, pre-mission testings, optimizations, modifications, preparation of all liquids and cell solutions, and for the on orbit and on ground experiments. The USOCs, BIOTESC and MUSC were responsible for the operational planning and the operational products, the BIOLAB telecommunication and remote control, as well as for communications with NASA and the astronauts during the on orbit operations. The European Space Agency (ESA) coordinated between all teams. DLR and NASA participated in the experiment preparation. During the preparation phase of the experiment, teleconferences with all parties were performed at a weekly basis. All planned experiment steps had to be approved and authorized by all parties before the scientists were allowed to conduct specific experiments in the prepration and testing phase. The complete experiment requirements were recorded in the Experiment Scientific Requirements (ESR) document.

5 Abbreviations

AAS	Automatic Ambient Stowage
AEC	Advanced Experiment Container
ATCS	Automatic Temperature Controlled Stowage
BGB	Biolab Glovebox
BIOTESC	Biotechnology Space Support Center
EC	Experiment Container
ELB	Extra liquid bag
EPU	Experiment Preparation Unit
ESR	Experiment Scientific Requirements
ESA	European Space Agency
HM	Handling Mechanism
HMPU	Handling Mechanism Plate Unit
IFB	Internal Filling Bag
ISPR	International Standard Payload Rack
MB	Measurement Bag
MELFI	Minus Eighty Laboratory Freezer for ISS
MUSC	Microgravity User Support Center
PMT	Photomultiplier Tube
SCB	Stock Culture Bag
TCU	Temperature Controlled Unit
TPLX-A	TRIPLE LUX A
USOC	User Support and Operations Center
